# Investigation of Cognitive Improvement in Alcohol-Dependent Inpatients Using the Montreal Cognitive Assessment (MoCA) Score

**DOI:** 10.1155/2016/1539096

**Published:** 2016-12-01

**Authors:** Stéphanie Pelletier, Bertrand Nalpas, Régis Alarcon, Hélène Rigole, Pascal Perney

**Affiliations:** ^1^Service Addictologie, Hôpital du Grau du Roi, CHU Caremeau, Nîmes, France; ^2^Département d'Information Scientifique et de Communication (DISC), Inserm, Paris, France; ^3^Université Montpellier I, Montpellier, France; ^4^Service de Médecine Interne et Addictologie, Hôpital Saint Eloi, Montpellier, France; ^5^Inserm U1178, Paris, France

## Abstract

*Background*. Cognitive dysfunction is a common feature in alcohol use disorders. Its persistence following alcohol detoxification may impair quality of life and increase the risk of relapse. We analyzed cognitive impairment changes using the Montreal Cognitive Assessment (MoCA) score in a large sample of alcohol-dependent inpatients hospitalized for at least 4 weeks.* Method*. This was an observational longitudinal survey. Inclusion criteria were alcohol dependence (DSM-IV) and alcohol abstinence for at least one week. The MoCA test was administered on admission and at discharge.* Results*. 236 patients were included. The mean MoCA score significantly increased from 22.1 ± 3.7 on admission to 25.11 ± 3.12 at discharge. The corresponding effect-size of improvement was high, 1.1 [95% CI 1.0–1.2]. The degree of improvement was inversely correlated with the baseline MoCA score. The rate of high and normal, that is, >26, MoCA values increased from 15.8% on admission to 53.8% at discharge. MoCA score improvement was not correlated with the total length of abstinence prior to admission.* Conclusion*. The MoCA score seems to be a useful tool for measuring changes in cognitive performance in alcohol-dependent patients. A significant improvement in cognitive function was observed whatever the degree of impairment on admission and even after a long abstinence period.

## 1. Introduction

Chronic excessive alcohol consumption is associated with numerous medical and psychological complications. Among them, cognitive dysfunction is the most frequent, ranging from mild impairment to irreversible damage such as the Korsakoff syndrome [1]. Numerous cognitive functions are impaired in alcohol-dependent patients, particularly executive functions [2], visuospatial skills [3], and episodic and working memory [4, 5]. The frequency of cognitive disorders in alcohol-dependent inpatients is reported to be high, ranging from 50 to 70% [6, 7]. This high prevalence is probably related at least in part to the duration of alcohol intoxication before seeking treatment.

Recovery from these cognitive disorders following alcohol withdrawal has been studied by several authors [8–11]. Using various neuropsychological tests, most authors found that visuospatial capacity, memory, and executive function did improve with continued abstinence, thus confirming what has been reported using neuroimaging approaches [12, 13]. However, the duration of abstinence required for normalization of cognitive function remains a matter of debate. Some studies reported a significant improvement after two to four weeks [14] of abstinence, whilst others did not find any modification at week 7 [15]. Moreover, reversibility may be different from one type of cognitive function to another since it depends on the rate of recovery of each brain area [16]. This rate of recovery is crucial for the patient's participation in relapse prevention programs. Alcohol-related cognitive defects may lead to poor participation in therapeutic workshops or absence of recording of therapeutic advice [17], thus impairing the global efficacy of rehabilitation programs. Another key factor in recovery is preservation of complete abstinence, although recent neuroimaging data suggest that certain brain regions might recover even if patients resume drinking small amounts [18]. As any lapse or relapse is difficult to detect and as report of abstinence is typically based on declarative or self-report data, interpretation of longitudinal studies of outpatients requires caution. Studies involving inpatients are preferable since they make it possible to more confidently assess abstinence-related cognitive recovery. Finally, there is a need for a simple, sensitive, and specific test if cognitive assessment is to be carried out systematically in routine practice. Several tests are available [19] but they assess either a single domain or several domains not modified in alcohol-dependent populations. They are lengthy to administer and often complicated; for example, in the study performed by Davies et al., administration lasted from 60 to 90 min [20], whilst Glass et al. [21] used 6 different tests. A new test, the Bearni, has been recently proposed [22] but was not available at the time we began our investigation.

It was recently reported that the Montreal cognitive assessment (MoCA) test is a convenient tool to screen for cognitive impairment in alcohol use disorders [23]. Therefore, it is relevant to evaluate whether the MoCA test could also be used to assess the changes in cognitive function following alcohol detoxification. In this regard, an interesting result was obtained by Likhitsathian et al. [24] but his study population was small (*n* = 38) and abstinence could not be guaranteed since included patients were not hospitalized throughout the entire observation period. For our purpose, we designed a longitudinal study aiming to evaluate, using the MoCA test, the changes in cognitive defects from admission to discharge in a large group of alcohol-dependent inpatients in rehabilitation for 4 to 8 weeks.

## 2. Patients and Methods

### 2.1. Patients

The study was conducted in a hospital-based substance use disorder rehabilitation center. Patients admitted to the center had to be free of any active drug intoxication and came either after a stay in hospital for detoxification or directly from their homes. The length of abstinence was not a decisive criterion for admission and could range from a few days to several weeks. Inclusion criteria in the current study were admission for dependence on alcohol assessed by the DSM IV; age above 18 years; ability to understand and speak French; oral agreement to participate; MoCA score < 26 at baseline or ≥26 despite a deeply impaired subscore. Exclusion criteria were severe comorbid neurologic or psychiatric disease such as dementia, Alzheimer's disease, and psychosis, past history of stroke or coma, encephalopathy, and refusal to participate.

We recorded sociodemographic data: age, sex, marital status (single/in a relationship), education level (less than 12 years, equal to or higher than 12 years), family history of alcohol/drug use disorders through a family tree, and smoking status. The clinical data were diagnosis of cirrhosis (yes/no, according to clinical examination, ultrasonography, and routine liver function tests) and treatment with benzodiazepine. Alcohol consumption was evaluated by the TimeLine FollowBack method [25] and dependence on tobacco by the Fagerstrom test [26]. Cannabis, cocaine, and heroin consumption were based on declarative data and urinary tests.

The recruitment period started in November 2012 and closed in September 2014.

#### 2.1.1. Rehabilitation Treatment

We did not aim to examine the effect of neuropsychological rehabilitation on cognitive recovery, so all the patients studied benefited from the same rehabilitation program and we did not include a control group.

The rehabilitation treatment program consisted in a set of activities common to all the patients, supervised either by a physician, a psychologist, or a nurse and performed in groups. These included information about alcohol-related disease and dependence and sleep disorders, sensitization to quitting tobacco, and physical training. Cognitive behavioral therapy sessions focusing on communication and social skills, popular belief about alcohol, identification of high-risk situations, and coping skills training. Patients were asked to attend self-help groups (Alcoholic Anonymous and others) which were held once a week. Each patient benefited from a weekly individual session with a psychologist.

Any alcohol consumption is completely prohibited in the unit, and no alcohol-containing beverages are sold within the hospital. Patients are aware that alcohol breath-tests are performed at random during their hospital stay. Exit from the hospital for personal convenience is allowed once a week and lasts 1.5 hours at most; an alcohol breath-test is performed when the patient returns.

### 2.2. Methods

We used the 7.1 version of the MoCA test translated into French provided by the MoCA test organization (http://www.MoCAtest.org/). The MoCA test was administered by experienced occupational therapists or neuropsychologists familiar with the test. They all used a similar scoring grid defined in accordance with the guidelines proposed [27]. The MoCA test was scheduled to be administered within the first week following admission and the day of or the day preceding discharge. The MoCA test was performed in a quiet room in the morning. If the patient was a smoker he/she was asked to refrain from smoking during the 30 min preceding the test to avoid any bias related to the acute effect of nicotine [28]. At discharge patients were asked whether they remembered their answers to the test administered at admission or not.

The MoCA score explores 8 cognitive domains: visuospatial/executive ability, naming, memory (not scored), attention, language, abstraction, delayed recall, and orientation.

The initially proposed [27] normal MoCA score is ≥26. However, this cut-off is debated in the literature since age, education level, cultural origin, and the screening conditions of the population studied [29] appear to influence the results of the test [30, 31]. According to the author who promoted the MoCA score, one point should be added to the total score in subjects with a low (<12 years) education level [27], although this was contradicted by Gagnon et al. [32]. As our present study aimed to evaluate the changes in cognitive performance using the MoCA score, we decided to use the raw MoCA score without any correction as we did in our previous study [23] and we kept 26 as the cut-off normal value in accordance with other authors [33, 34].

Variation in the MoCA score was analyzed as the absolute difference between the MoCA score at discharge minus the score on admission. In a secondary analysis we also calculated the relative improvement. Given that the maximal MoCA score is 30, we calculated the maximal percent increase (MPI) for each patient using the following formula: (maximal MoCA score − MoCA score on admission/MoCA Score on admission) *∗*100; for example, a patient with a MoCA score equal to 10 on admission could increase the score by (30 − 10/10) *∗* 100 = 200%; then we calculated the actual percent increase (API) and compared it to the MPI.

### 2.3. Ethics

Our study was an observational study without any intervention in the care of the patients. According to French law, no formal written consent is required in such cases. However, patients were orally informed of the study.

### 2.4. Statistics

Quantitative values were described using their number, mean, standard deviation, and median; MoCA scores on admission and discharge were compared using ANOVA with repeated measures, with age and education as covariables. Qualitative values were described using their number, frequencies, and centiles and were compared using the Chi^2^ test or Fisher's exact test when necessary. To evaluate the change in MoCA score as a function of its baseline value, we split the study population into three subgroups with low (≤21), intermediate (≥22 and ≤25), and high (≥26) MoCA scores. All analyses were performed using SPSS software V22.0 (IBM SPSS Inc., Armonk, NY, USA).

## 3. Results

During the study period, 236 patients admitted for rehabilitation after alcohol withdrawal met the inclusion criteria and were evaluated using the MoCA test on admission and at discharge. Their main characteristics are presented in Table 1. There were 162 men (68.6%) and 74 women (31.4%) aged 50.2 ± 9.6 and 50.9 ± 9.4 years, respectively. There were no differences between men and women, except for the quantity of alcohol consumed which was slightly but significantly higher in the former than in the latter (214 ± 135 vs 182 ± 123 g/d, *p* = 0.04). Most patients (82.2%) were tobacco smokers and none of them stopped smoking following admission (smoking is forbidden inside the hospital but allowed outside); 21.2% regularly smoked cannabis but had stopped their consumption on admission as smoking cannabis is prohibited during the rehabilitation stay. About two-thirds of the patients (66.9%) had a low educational level (<12 years), 14.8% had an average level (12 years). and the remaining 18.2% had a high level (>12 years). Two-thirds (65.7%) were occupationally inactive, including 17.3% who were retired.

About one-half of the patients (48.7%) had a family history of alcohol misuse. Mean alcohol consumption was 204 ± 132 g of pure alcohol per day. The patients had been drinking excessively for 14.9 ± 10.3 years. One hundred and thirty-two patients (56%) drank wine and/or beer only and 103 (44%) drank hard liquors (whiskey, bourbon, etc.) with or without wine or beer. Forty-three patients (18.4%) had cirrhosis, but none displayed any clinical sign (depressed level of consciousness or flapping tremor) of hepatic encephalopathy.

### 3.1. MoCA

The mean duration of MoCA test administration was approximately 20 min. The median duration of abstinence from alcohol at the time of MoCA administration was 21 days (25th–75th percentiles: 14–56 days). As diazepam is currently used for alcohol withdrawal, we checked benzodiazepine (bzd) status on the day of MoCA administration: 38.6% of the patients were free of any bzd administration even before admission, 31.5% were in the late phase of the bzd treatment given for alcohol withdrawal, and 16.2% and 13.6% were treated chronically (i.e., before admission) by low (<20 mg) or high (>20 mg) doses, respectively.

### 3.2. Admission

On admission, the mean MoCA value was 22.1 ± 3.7 (percentiles 25th–75th : 20–25) and 84.2% of patients had a value lower than 26. Eighty-eight patients (37.3%), 111 (47.0%) and 37 (15.7%) belonged to the low, intermediate, and high MoCA score, respectively. Analysis of the MoCA subscores showed that five domains were specifically impaired, namely, visuospatial capacity, attention, language, abstraction, and delayed recall (Table 2).

There was a weak, although significant (*p* < 0.05) correlation between MoCA value and age analyzed as a continuous variable and between MoCA value and education level (<12 versus >12 years, *p* = 0.05). Conversely, the mean MoCA value did not vary according to sex, or to the number of previous alcohol detoxifications, or to family history of alcoholism, or to the time elapsed between alcohol withdrawal and admission even after stratification on education levels.

### 3.3. Discharge

At discharge, which occurred 33.5 ± 7 days after admission, most patients roughly remembered the nature of the test but not the answers they had given. The MoCA score was significantly increased compared to baseline values (25.11 ± 3.12 versus 22.15 ± 3.70, *p* < 0.001), the mean gain being 3 points for the whole sample (Table 2), and the corresponding effect-size of improvement was high, 1.1 [95% CI 1.0–1.2]. Analysis stratified on the baseline MoCA scores showed that improvement occurred in all three groups with a mean increase of 4.2, 2.6, and 0.9 in the groups with low, intermediate, and high MoCA scores at baseline, respectively. The corresponding effect-size of improvement was high, 1.6 and 1.2 in the groups with low or intermediate MoCA scores at baseline, respectively, and medium, that is, 0.6, in the group whose score was ≥26 on admission (Table 3).

**Figure 1:**
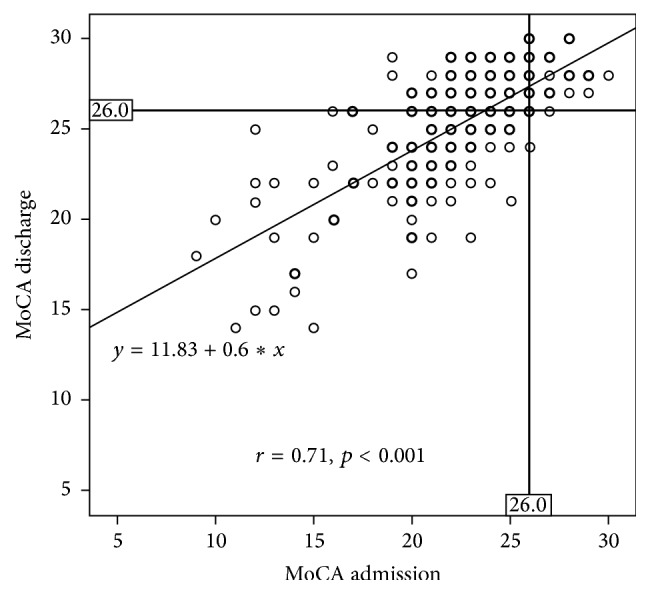
Plot of MoCA scores on admission against MoCA scores at discharge.

MoCA scores on admission and at discharge were highly correlated (*r* = 0.71, *p* < 0.001) and the linear regression line was *y* = 11.83 + 0.6*x* (Figure 1).

Detailed analysis of the MoCA subscores showed that the five domains which were impaired on admission improved significantly (*p* < 0.001), specifically attention and delayed recall which increased by 1.45 and 0.95 points, respectively (Table 2).

The per patient analysis showed that the MoCA score increased in 200 patients, plateaued in 18 patients, and decreased in the remaining 18 patients. The increase ranged from 1 (in 30 patients) to 13 points (in 1 patient). The decrease was equal to 1, 2, 3, and 4 points in 10, 5, 1, and 2 patients, respectively; the loss mainly concerned the domains visuospatial ability, attention, and recall (data not shown).

On admission, 37 patients (15.8%) had a high MoCA score, that is, ≥ than 26, but at discharge the number had increased to 127 (53.8%). Analysis according to MoCA score at baseline showed that among the patients in the lower group on admission, up to 20% had moved to the high group at discharge (Table 4), and 54.5% had progressed to the intermediate group; among the 7 remaining patients (27.3%), 2 remained stable, and 3, 1, and 1 lost 1, 2, and 3 points, respectively. In the patients belonging to the intermediate group on admission, more than two-thirds (67.6%) were in the high group at discharge and about 30% plateaued. Finally, all the patients with a high score at admission except one were still in their baseline group at discharge.

The relative benefits were analyzed according to the categories of MoCA score on admission. In the subgroup with a low MoCA score, the API was 25.2 ± 22.7% for a MPI equal to 68.4 ± 38.9%; the corresponding figures for the intermediate subgroup were 11.4 ± 9.4% (API) and 28.1 ± 6.2% (MPI) and finally 3.7 ± 6.0% (API) and 11.7 ± 45% (MPI) for those who had a high MoCA score on admission.

On the whole, the mean potential maximal gain for the entire group was 41.6 ± 33.2% (median 36.3%). At discharge, the mean actual gain was 17.2 ± 16.7% (median 13.6%).

### 3.4. Variables Associated with MoCA Score Improvement

Improvement in MoCA scores was not different according to sex, smoking status, cannabis consumption, presence of cirrhosis, family history of alcoholism, treatment with benzodiazepine, daily alcohol consumption, duration of excessive drinking, or the length of prior abstinence before admission, although this variable was close to the significance limit. Conversely, it was significantly associated with age (*p* < 0.001) and education level (*p* < 0.001), although the correlation coefficients were moderate (0.17 and 0.27, respectively).

## 4. Discussion

Whilst the harmful effect of chronic excessive alcohol consumption on cognition is well-known, evaluating the improvement of impaired functions following abstinence is rather complicated for several reasons. The study population should be large owing to the high variability in the degree of cognitive impairment from one patient to another, the patients should be hospitalized in order to control abstinence, and the tool used to assess variation in cognitive dysfunction should be easy to understand by all patients and simple to administer. Our work met these requirements. We included 236 patients who were in a controlled alcohol-free environment since they were hospitalized in a rehabilitation treatment center for several weeks and we assessed cognitive improvement using a validated tool, the MoCA test.

The Montreal cognitive assessment (MoCA) test was proposed in 2005 for the evaluation of cognitive deficits [27], and its psychometric properties were reevaluated recently in a large population [35]. It is now currently used for several conditions. A search for “MoCA test” in PubMed retrieved 330 articles (access on June 17, 2016) and we recently reported its usefulness in an alcohol-dependent population [23].

In this study, we evaluated the variation of MoCA scores according to time in abstinent patients. As the same questionnaire was administered on admission and at discharge, a possible test-retest effect should be taken into account. However, the effect, if any, should be low since it has been shown that test-retest performance is very good even at one month with no significant learning effect [27]. Furthermore, none of the patients remembered the answers they had given previously.

On admission, in terms of age and sex ratio, the patient sample had the typical sociodemographic profile of alcohol-dependent people (or with severe alcohol use disorder according to the new DSM5 classification) seeking therapy in our geographical area [23, 36, 37].

The global MoCA score was close to that observed in a previous work [23]. The cognitive domains which were the most impaired were visuospatial capacity, attention, language, abstraction and delayed recall, in accordance with other studies [3, 20, 38, 39].

At the end of the rehabilitation program, cognitive function had strongly improved, as observed by the large effect-size of the difference in MoCA score on admission and discharge. The domains attention, recall, and visuospatial ability were significantly better. These results are consistent with those of Likhitsathian et al. [24], although the degree of improvement was higher in our study. The rates of absence of change and decrease in MoCA scores were low, and in the event of decreased scores, worsening was slight. However, patients with a low MoCA score on admission who did not improve during rehabilitation may require further neurological investigations and we intend to verify this hypothesis in the near future. Of course, improvement was a function of initial impairment, but a substantial and significant benefit was observed even in those patients with only slight dysfunction at baseline.

The normal MoCA score is reported to depend on age and education level as well as the Mini Mental State Examination (MMSE) [40] but the precise cut-off for a given age and education level are yet to be properly defined. That is why, when we split the population in our study, we named the subgroups as having low, intermediate, or high MoCA scores rather than presenting with severe, moderate, or no cognitive impairment. However, as the maximal MoCA score is 30, one could consider that the closer a given subject's result is to this value, the better the patient's cognitive function is; therefore, although a cut-off equal to 26 might not be the “normal” value for everybody, a MoCA score equal to or above this value indicates that cognitive function is highly efficient.

One potential value of the MoCA test is that it can be used to globally evaluate cognitive function which is finally summarized in a score with its own cut-off of normality. Our results showed that the rate of patients with high MoCA scores, corresponding to efficient cognitive function, was more than three times higher at discharge than on admission. Of course this does not demonstrate the efficacy of the therapeutic program since our protocol was not designed with this aim and cognitive improvement might well be due to alcohol abstinence only as reported in other studies [8, 9, 11]. However, it should be noted that neither the MoCA score on admission nor the actual gain during the hospital stay differed according to the length of alcohol abstinence prior to admission. As the long-term abstinent patients included in this study came from their homes, this suggests that being abstinent in a nonstimulating environment could alleviate cognitive deficits, but only to a limited degree, a hypothesis which should be confirmed. Further progression towards complete improvement would require both abstinence from alcohol and a stimulating environment, two conditions which are found in some specialized rehabilitation treatment centers. In this regard, analysis based on percent increase in MoCA scores clearly shows that at the end of the rehabilitation program, there is still room for progression.

## 5. Conclusion

In conclusion, the MoCA test seems to be a useful tool for evaluating changes in cognitive function in alcohol-dependent patients during a stay in a rehabilitation treatment center. A significant improvement appears to occur whatever the degree of cognitive impairment and even after a long abstinence period. Conversely, the absence of improvement may be the indication for further neurological investigations.

## Figures and Tables

**Table 1:** Main characteristics of the patients studied.

	Male	Female	Total	*p*
*N*	162	74	236	
Age (M ± SD)	50.2 ± 9.6	50.9 ± 9.4	50.4 ± 9.5	NS
Education level (%)				NS
<12	71.6	56.8	66.9	
12	11.7	21.6	14.8	
>12	16.7	21.6	18.2	
	100	100	100	
MoCA score at admission	21.9 ± 3.9	22.7 ± 3.2	22.1 ± 3.7	NS
Smokers (%)	82.1	82.4	82.2	NS
Cannabis (%)	21	21.6	21.2	NS
Alcohol quantity (g/d)	214 ± 135	182 ± 123	204 ± 132	0.04
Duration (years)	14.3 ± 10.4	14.1 ± 9.0	14.9 ± 10.3	NS
Family history of alcohol misuse (%)	46.3	54.1	48.7	NS

**Table 2:** MoCA scores and subscores on admission and discharge in the 236 patients studied.

	MOCA scores total	MoCA subscores						
		Visuospatial	Naming	Attention	Language	Abstraction	Delayed recall	Orientation
Max score	30	5	3	6	3	2	5	6

Admission	22.15 ± 3.70	2.94 ± 1.41	2.94 ± 0.23	3.62 ± 1.45	2.11 ± 0.70	0.83 ± 0.69	3.04 ± 1.40	5.55 ± 0.74
Discharge	25.11 ± 3.12^1,2^	3.75 ± 1.06^1^	2.96 ± 0.19	5.07 ± 1.17^1^	2.42 ± 0.63^1^	1.05 ± 0.77^1^	3.99 ± 1.24^1^	5.84 ± 0.41^1^

^1^ *p* < 0.001 versus discharge; ^2^effect-size: 1.1 [95% CI 1.0–1.2].

**Table 3:** MoCA scores on admission and at discharge and effect-size of the difference in the sample divided into three subgroups with increasing MoCA scores.

MoCA subgroups on admission	*N*	MoCA scores		*p*	Effect-size	95% CI	
		Admission	Discharge			Lower	Upper
Low (≤21)	88	18.5 ± 3.1	22.7 ± 3.2	<0.001	1.4	1.2	1.6
Intermediate (22–25)	111	23.5 ± 1.1	26.1 ± 1.9	<0.001	1.2	1.1	1.4
High (≥26)	37	26.9 ± 1.1	27.8 ± 1.3	0.01	0.6	0.3	0.9
All	236	22.1 ± 3.7	25.1 ± 3.1	<0.001	1.1	1.0	1.2

**Table 4:** Changes in MoCA subgroup distribution from admission to discharge.

MoCA subgroups on admission	*N*	MoCA subgroups at discharge (%)		
		Low (≤21)	Intermediate (22–25)	High (≥26)
Low ≤21	88 (37.2%)	27.3	54.5	18.2
Intermediate (22–25)	111 (47.0%)	2.7	29.7	67.6
High (≥26)	37 (15.8%)	0.0	2.7	97.3

All	236 (100%)	11.4	34.7	53.8
